# Antimicrobial effects of airborne acoustic ultrasound and plasma activated water from cold and thermal plasma systems on biofilms

**DOI:** 10.1038/s41598-020-74504-z

**Published:** 2020-10-14

**Authors:** Clémentine M. G. Charoux, Apurva D. Patange, Laura M. Hinds, Jeremy C. Simpson, Colm P. O’Donnell, Brijesh K. Tiwari

**Affiliations:** 1 Department of Food Chemistry and Technology, Teagasc Food Research Centre, Ashtown, Dublin, Ireland; 2 School of Biosystems and Food Engineering, University College Dublin, Dublin, Ireland; 3 School of Biology and Environmental Science, University College Dublin, Dublin, Ireland

**Keywords:** Microbiology, Bacteria

## Abstract

Bacterial biofilms are difficult to inactivate due to their high antimicrobial resistance. Therefore, new approaches are required for more effective bacterial biofilm inactivation. Airborne acoustic ultrasound improves bactericidal or bacteriostatic activity which is safe and environmentally friendly. While, plasma activated water (PAW) is attracting increasing attention due to its strong antimicrobial properties. This study determined efficacy of combined airborne acoustic ultrasound and plasma activated water from both cold and thermal plasma systems in inactivating *Escherichia coli* K12 biofilms. The application of airborne acoustic ultrasound (15 min) alone was significantly more effective in reducing *E. coli* counts in 48 and 72 h biofilms compared to 30 min treatment with PAW. The effect of airborne acoustic ultrasound was more pronounced when used in combination with PAW. Airborne acoustic ultrasound treatment for 15 min of the *E. coli* biofilm followed by treatment with PAW significantly reduced the bacterial count by 2.2—2.62 Log_10_ CFU/mL when compared to control biofilm treated with distilled water. This study demonstrates that the synergistic effects of airborne acoustic ultrasound and PAW for enhanced antimicrobial effects. These technologies have the potential to prevent and control biofilm formation in food and bio-medical applications.

## Introduction

Biofilms are an assemblage of surface-associated microbial cells that are enclosed in a self-produced extracellular polymeric substance matrix; which is composed of polymeric conglomeration of extracellular polysaccharides, proteins, lipids and DNA^1^. Due to the complexity of their structure, biofilms exhibit higher antibiotic resistance to disinfection processes than planktonic cells^2^. Biofilms can form on both biotic and abiotic surfaces and are prevalent throughout industrial environments^3,4^. Various techniques for removal of the clustered cells exist, mostly involving chlorinated sanitizers, organic acids and other chemical products. However, most of the chemical agents either require high concentration or are ineffective to penetrate into the whole biofilm structure^5,6^. While, some chemical agents like chlorine based products have resulted in health and environmental issues associated with chemical residues and production of toxic by-products^7^. Poorly maintained equipment, unhygienic conditions, and improper cleaning procedures in food manufacturing environments may increase the tolerance of microbial biofilms to disinfectants. Bacterial populations are known to carry genes that may protect the bacteria under adverse conditions. For instance, the presence and distribution of *qacH* and *bcrABC* genes in *Listeria monocytogenes* ensure survival and growth of this strain when subjected to sub-lethal levels of disinfectants based on quaternary ammonium compounds^8^.

Food contact surfaces are a major source of food product microbial contamination during processing, transport and storage. Stainless steel is a common food contact surface for biofilm adhesion in the food industry^9^. Airborne acoustic ultrasound employs non-contact transducers which are capable of transmitting ultrasonic waves to the product using air as the coupling medium^10^. In recent years, the design of airborne acoustic ultrasonic devices has been significantly advanced and successfully demonstrated in various food industry applications including; drying, defoaming and decontamination. However, to date, it has not been employed for microbial biofilm disruption.

Plasma is defined as a partially or wholly ionized gas composed of positive and negative ions, electrons, photons, free radicals and neutrons atoms and molecules^11^. Plasma can be categorised into thermal (equilibrium) and non-thermal (non-equilibrium) plasma^12^. Thermal plasma has all constituent species, electrons, ions at same temperature, thus in thermodynamic temperature equilibrium state. Whereas, non-thermal plasma is characterised by higher temperature of electrons compared to other active species within plasma i.e. they are in non-equilibrium state^13^. The cooling effect of ions and uncharged particles are much effective than energy transfer from electrons, therefore the gas still remains in low temperature, therefore they are also referred as cold plasma^13^. Plasma has been mainly used in bio-medical, textile and polymer industries^14–16^. However, recent studies have demonstrated potential applications of plasma technology in the food industry^17,18^. There is a range of different approaches to generate plasma discharges, using different geometry configuration. Typical corona discharge, dielectric barrier discharge (DBD), radio-frequency (RF) discharge, plasma jet (APPJ), microwave discharge are commonly used. Recently, atmospheric plasma jet is widely used plasma discharge, which uses air and special electrode design to prevent arcing. Due to its design and configuration, plasma jet has been applied for biomedical^19^, surface decontamination^20^ or food system^21^. Atmospheric plasma jet is an efficient technology with high chemical activity that inactivates wide range of microorganisms^20,22^.

There are a range of different methods to apply plasma treatment on the targeted product: direct or indirect mode of plasma exposure^23^. Several studies have applied atmospheric pressure plasma systems directly to biofilms^24^. In the last few years, plasma treated liquids have also shown promising effect. Plasma can also be used indirectly via plasma activated water (PAW), whereby the targeted product is not subjected directly to the plasma gas, but is in contact with water which was pre-treated with plasma. Direct treatment has some limitations, including effects on food quality and changes in surface topography. Treating biofilms via PAW is an alternative to direct contact, which can overcome these limitations for water-soluble products^25^. Replacing the chemical liquid products used in the food industry to remove biofilms with PAW rich in reactive species would be simple to integrate into industrial cleaning protocols. The objective of this study was to investigate the inactivation efficacy of individual and combined airborne acoustic ultrasound and PAW treatments from both cold and thermal plasma systems on *Escherichia coli* K12 biofilms.

## Results and discussion

### Effect of airborne acoustic ultrasound on bacterial biofilms

**Figure 1:**
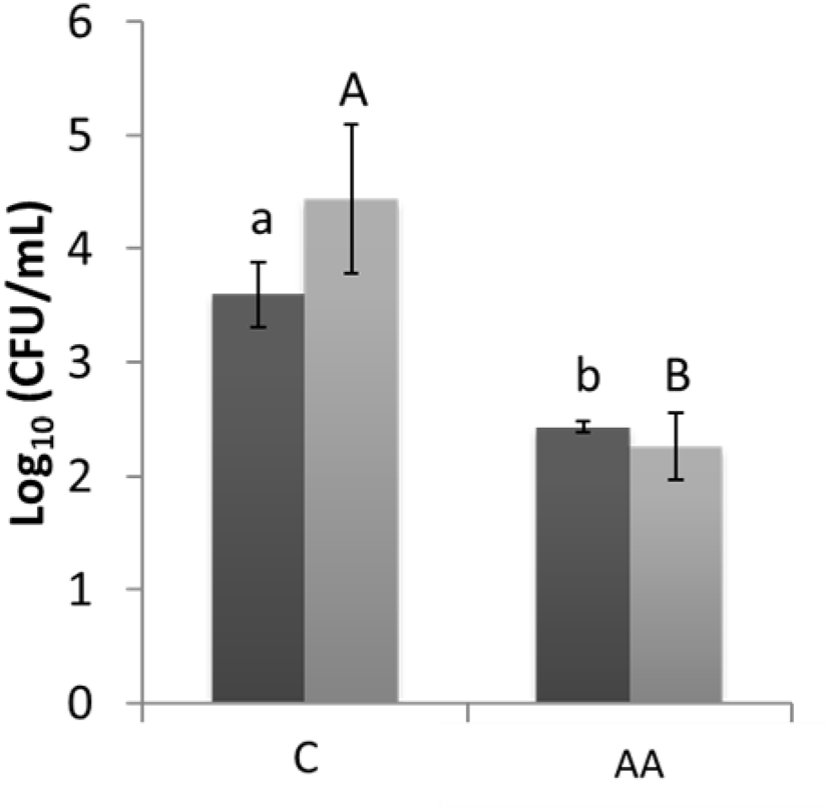
Microbial count after biofilm detachment of ultrasonic-treated samples. “Black square” 48 h biofilm “Grey square” 72 h biofilm. C: control; AA: Airborne acoustic ultrasound 15 min. ^a,b^Different letters on column are significantly different (*p* < 0.05). ^A, B^ Different letters on column are significantly different (*p* < 0.05). Error bars stand for standard deviation.

Figure 1 shows the Log_10_ CFU/mL of biofilms grown for 48 and 72 h on stainless steel coupons and treated using airborne acoustic ultrasound for 15 min. A slightly higher number of adherent cells were observed in case of untreated controls after 72 h of incubation compared to 48 h. Regardless of the higher number of cells, significant reductions of 1.17 ± 0.24 Log_10_ CFU/mL and 2.19 ± 1.01 Log_10_ CFU/mL were observed for treatment of samples grown for 48 h and 72 h respectively (*p* < 0.05). This significant reduction in biofilms after airborne acoustic ultrasound treatment can be attributed to morphological changes occurring in bacterial cell membranes. Previous studies demonstrated fragmented and ruptured bacterial cells with 15 min airborne acoustic ultrasound treatment^26^. Ultrasound creates stable cavitation and micro-streaming which causes direct damage to the ultrastructure of bacterial cells in biofilms^27^. However, the acoustic intensity used in this study is relatively much lower compared to contact power ultrasound systems. The microbial inactivation due to airborne acoustic ultrasound can mainly be attributed to physical effects caused by acoustic pressure and partly due to thermal effects and ultrasonic wave stresses^28^. However, the exact mechanism of microbial inactivation for non-contact type ultrasound is not yet fully understood.

### Antimicrobial efficacy of plasma activated water treatment and synergistic effects with airborne acoustic ultrasound

**Figure 2:**
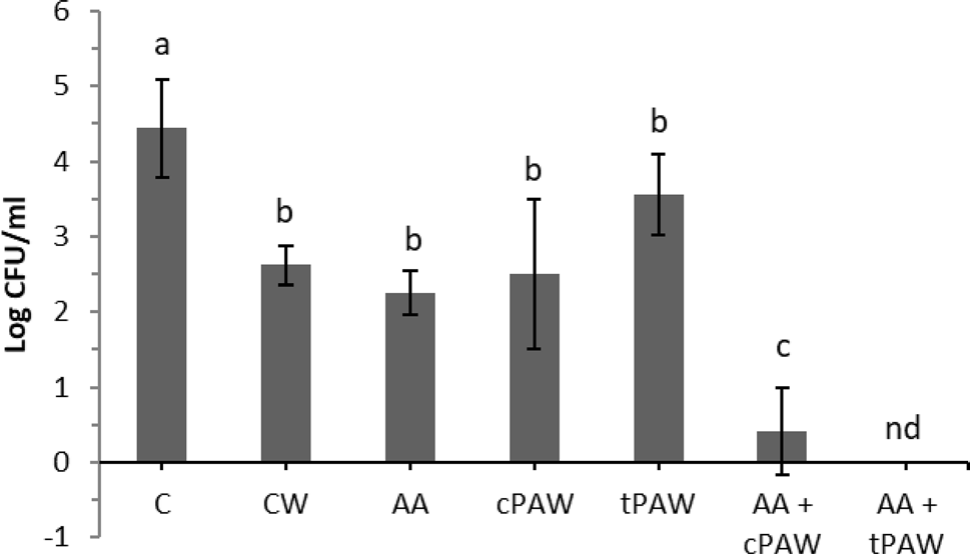
Microbial count after (72 h) biofilm detachment of samples treated with cold and thermal plasma-treated-water alone and in combination with ultrasound treatment. ^a,b,c^Different letters on column are significantly different (*p* < 0.05); ‘nd’ indicates “not detected”; Error bars stand for standard deviation.

The effect of PAW alone and in combination with airborne acoustic ultrasound was assessed on 72 h *E. coli* biofilms formed on the stainless-steel surfaces. Figure 2 presents the Log_10_ CFU/mL of biofilms grown for 72 h and treated with either cold or thermal PAW alone and in combination with airborne acoustic ultrasound. It can be observed that dipping a biofilm inoculated coupon into non-treated water (CW) significantly reduced the cell population from 4.44 ± 0.65 Log_10_ CFU/mL to 2.62 ± 0.25 Log_10_ CFU/mL. The reduction in microbial counts for biofilms washing with water alone is mainly due to the weak biofilm formation of the selected microorganism. The biofilm coupons noted as ‘C’ received no treatment; ‘CW’ were treated with sterile distilled water; ‘cPAW’ were treated with PAW generated from cold plasma; ‘tPAW’ were treated with PAW generated from thermal plasma; ‘AA’ were treated with airborne acoustic ultrasound; ‘AA + cPAW’ were treated with airborne acoustic ultrasound and PAW generated from cold plasma; ‘AA + tPAW’ were treated with airborne acoustic ultrasound and PAW generated from thermal plasma. Biofilms treated with CW, cPAW and tPAW did not show any significant reductions in the count, whereas combined treatment of cold plasma generated PAW with airborne acoustic ultrasound showed a significant reduction of approx. 2.2 ± 0.59 Log_10_ CFU/mL when compared to control biofilm treated with distilled water. While, *E. coli* biofilm cells were completely reduced to undetectable levels when treated with AA + tPAW.

The inactivation of biofilms by a combination of PAW and airborne acoustic ultrasound is mainly due to the reactive species in PAW and the physical disruption of the biofilms due to ultrasound. The main biological effects induced due to plasma treatment are attributed to reactive species (reactive oxygen and nitrogen species)^29^. But biofilms by nature are complex biomaterials, (i) the biofilm architecture and (ii) its composition may limit penetration of reactive species into the biofilm matrix. Many previous studies demonstrated, that increasing treatment time and contact period could improve plasma inactivation efficacy against bacterial biofilm^30–33^. However, in this study PAW was combined with airborne acoustic ultrasound technology in order to obtain higher inactivation efficacies. An increase in the reduction factor was observed for the combined treatments, where exposure to airborne acoustic ultrasound significantly increased the susceptibility of bacterial biofilms to plasma treatment. These results are in agreement with the study by Yang et al.^34^, where a significant reduction in mature *Candida albicans* biofilms was observed when exposed to contact type ultrasound treatment in combination with antifungal agent Amphotericin B. Similarly, Wang et al.^35^ also demonstrated that low frequency ultrasound can enhance the activity of vancomycin against methicillin-resistant Staphylococcus aureus (MRSA) and methicillin-susceptible Staphylococcus aureus (MSSA) biofilms. However, the inactivation efficacy in these studies was influenced by antimicrobial/antifungal agents, drug concentration, holding and irradiation time.

The combined treatment of airborne acoustic ultrasound with PAW enhanced the antimicrobial efficacy of PAW. Regarding the inactivation mechanism, one plausible explanation is that airborne acoustic ultrasound disrupts extracellular polymeric substances, provoking lesions at the surface of the biofilm, which eases the penetration of the reactive species generated in water into biofilm matrix, causing higher inactivation. Recent review on PAW proposed that the antibacterial efficacy of PAW is mainly attributed to the synergistic effects of plasma reactive species, pH and oxidation–reduction potential (ORP)^25,36^. The exact details and mechanisms for plasma on biofilms are still poorly understood. The antimicrobial effect of PAW on bacterial biofilms are depended on the specifically involved plasma species, its composition, its interactions with biofilm matrix components and its diffusion into the biofilm cells^29^. Researcher have hypothesised that the interaction of plasma-induced reactive oxygen nitrogen species (RONS) with biofilm matrix components and solutes in the hydrated matrix could cause oxidation reaction as well as phosphodiesterase activity in cells which leads to physiological alterations and reduction in biofilm matrix area^36,37^. Eventually loss of matrix reduces the adhesiveness and dispersal of biofilm cells. While the biofilm is dispersing the remaining cells are exposed to RONS and become more susceptible to be inactivated by PAW. The reactive species generated in PAW not only affects the cell membrane integrity but also penetrate into membrane channels and provoke reactive oxygen species (ROS) production endogenously which account for the intracellular ROS accumulation inside the cell^31,38^. Studies by Lukes et al^39^ and Xu et al^31^ also demonstrated that RONS interact with intracellular components (DNAs, proteins, lipids) and influence its metabolic responses in microorganisms. These interactions cause oxidative stress on cell components thus initiating lipid and protein peroxidation on the cell membrane, followed by protein and/or DNA damage and cell death^36^.

**Figure 3:**
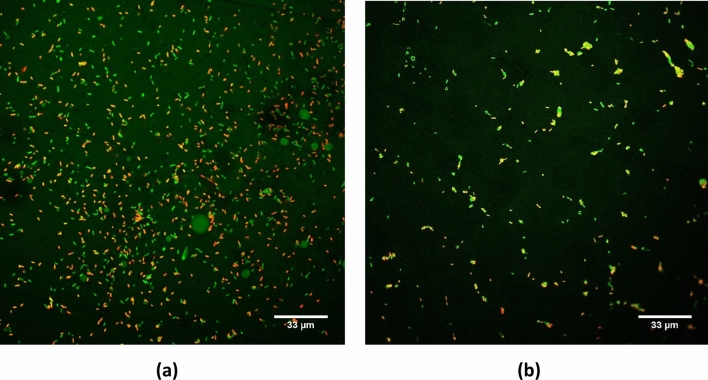
Confocal laser scanning microscope images of *E. coli* biofilm on glass slide (**a**) non-treated and following (**b**) 15 min treatment of airborne ultrasound. The cells were stained with STYO 9 (green fluorescence, live cells)/PI (red/yellow, dead cells). All images were compiled, and noise reduction filter was applied in the Olympus Fluoview FV1000 software (version 4.1.1.5. https://www.olympus-lifescience.com/en/support/downloads/ ).

The effect of these treatments on the biofilms was also observed using confocal microscopy. Confocal images of treated and control samples are shown in Fig. 3. The population of biofilms was significantly affected by the airborne acoustic ultrasound treatment. Following the same trend as the plate count method, the images have shown that airborne acoustic ultrasound is an effective tool for biofilm removal. The dead cells (red/yellow-fluorescent stained) increased upon treatment, thus indicating the bactericidal effect of airborne acoustic ultrasound alone or when used in combination with PAW.

### Physicochemical properties of plasma activated water

Table 1 shows that pH values of the PAW decreased significantly after cold or thermal plasma treatment. The pH of the solution is an important bactericidal property of PAW^40^; however the reduction in pH is not the sole cause for the bacterial inactivation^41^. The reason for acidification of PAW is predominantly due to the nitrate and nitrous acids generated by plasma treatment. Nitrates and nitrites in PAW are formed through dissolution of nitrogen oxides (NO_X_) formed in the air into water^36^. As demonstrated in Table 1, the concentration of nitrates and nitrites increased with plasma treatment, with higher levels of reactive nitrogen species (RNS) generated in cold PAW than thermal PAW.

**Table 1:** Nomenclature of different samples with conditions detailed.

Samples nomenclature*	Airborne acoustic ultrasound treatment	Cold plasma activated water treatment	Thermal plasma activated water treatment
C	–	–	–
CW	–	–	–
cPAW	–	30 min soaking	–
tPAW	–	–	30 min soaking
AA	15 min	–	–
AA + cPAW	15 min	30 min soaking	–
AA + tPAW	15 min	–	30 min soaking

*‘C’ were without any treatment; ‘CW’ were treated with sterile distilled water; ‘cPAW’ treated with cold plasma treated PAW; ‘tPAW’ treated with thermal plasma treated PAW; ‘AA’ treated with airborne acoustic ultrasound, ‘AA + cPAW’ treated with airborne acoustic ultrasound and cold plasma treated PAW; ‘AA + tPAW’ treated with airborne acoustic ultrasound and thermal plasma treated PAW.

The higher the oxidation ability of a solution to take electrons from cell membranes of the bacteria, the higher is the cell instability, which causes damage to the outer and inner cell membranes^42^. Both plasma treatment methods increased the ORP in the treated PAW in comparison to the control. H_2_O_2_ is considered to be a potent anti-microbial agent. Several studies have investigated about the importance of H_2_O_2_ to the antimicrobial activity of PAW^18,25^. In this study, concentration of H_2_O_2_ was generated in PAW. In addition, the conductivities of PAW were also recorded in this experiment. As shown in Table 1, the conductivity of PAW after plasma activation was significantly higher compared to the distilled water control. This is due to the generation of active groups (reactive oxygen and nitrate species) formed in water which improves the conductivity. Furthermore, the conductivity of PAW generated from cold plasma jet was higher than that generated from the thermal plasma jet. Overall, no significant difference was observed in the physicochemical properties of PAW generated from cold or thermal plasma, except for RONS concentration, which explains the similar inactivation efficacies observed.

## Conclusion

This study demonstrates the individual and combined antimicrobial effects of airborne acoustic ultrasound and PAW for the disruption and inactivation of biofilms. Airborne acoustic ultrasound is an effective tool for biofilm removal and PAW can inactivate cells due to enhanced diffusion of plasma activated water into the matrix. Shortcomings of current treatment approaches together with resistance among bacterial strains against key antimicrobial agents i.e. antibiotics could potentially be addressed through use of these novel technologies. Novel technologies such as plasma and airborne acoustic ultrasound have the potential to effectively eradicate persistent bacterial biofilms with less energy and short treatment times in food processing environments. However, further studies are required to better understand the inactivation mechanisms of these treatments, which affect the physiological and metabolic states of bacteria.

## Material and methods

### Preparation of coupons for biofilm cultivation

#### Stainless steel coupons

The surface material investigated in this study was grade 304 stainless steel; coupons (16 mm × 1.2 mm) were obtained from Watermark Engineering (Tallaght, Dublin 24, Ireland). Prior to inoculation, the coupons were placed in Duran bottles and sterilised at 121 °C for 15 min.

#### Glass slides

For confocal laser scanning microscopic examination, bacterial biofilms were grown on glass slides (1 × 1 cm). The glass slides were washed with acid (1 M HCl) followed by 70% ethanol as described by Fischer et al.^43^. After washing, the glass slides were air dried in fume hood on Whatman filter paper for 10–15 min before autoclaving at 121 °C for 15 min.

### Culture preparation and biofilm formation on coupons

Non-pathogenic *Escherichia coli* K12 ER2925 was obtained from the microbiological culture stock in Teagasc Food Research Centre (Ashtown, Dublin, Ireland). A loopful of *E. coli* K12 from the stock culture was transferred into nutrient agar plate (Product code: CM0003B; Oxoid Ireland c/o Fannin Healthcare, Ireland) and incubated at 37 °C for 24 h. A colony was then inoculated in 5 mL of Luria Bertani (noted LB, composed of 10 g of tryptone and 5 g of yeast extract per litre) and incubated for 24 h at 37 °C under shaking conditions (150 rpm). This culture was designated as a working culture and was stored at 4 °C until use, and a new culture was prepared weekly. On the day of the experiment, the optical density (O.D) was measured using EPOCH2C microplate reader (Biotek, Mason Technology Ltd, Dublin, Ireland) and adjusted to 0.15 using fresh LB media which corresponded to approx. 8 Log_10_ CFU/mL. Sterile coupons were placed in sterile 12-well plates and 1 mL of the OD-adjusted culture was added into each well. The 12-well plates were incubated at 37 °C for 48 and 72 h without medium change or agitation. After incubation, each coupon was washed twice using phosphate buffer saline BR0014 (PBS; Oxoid Limited, Ireland) to remove loosely attached and planktonic cells. The coupons were air dried inside a safety cabinet for 20 min.

### Airborne acoustic ultrasound and plasma activated water treatments

#### Airborne acoustic treatment

**Figure 4:**
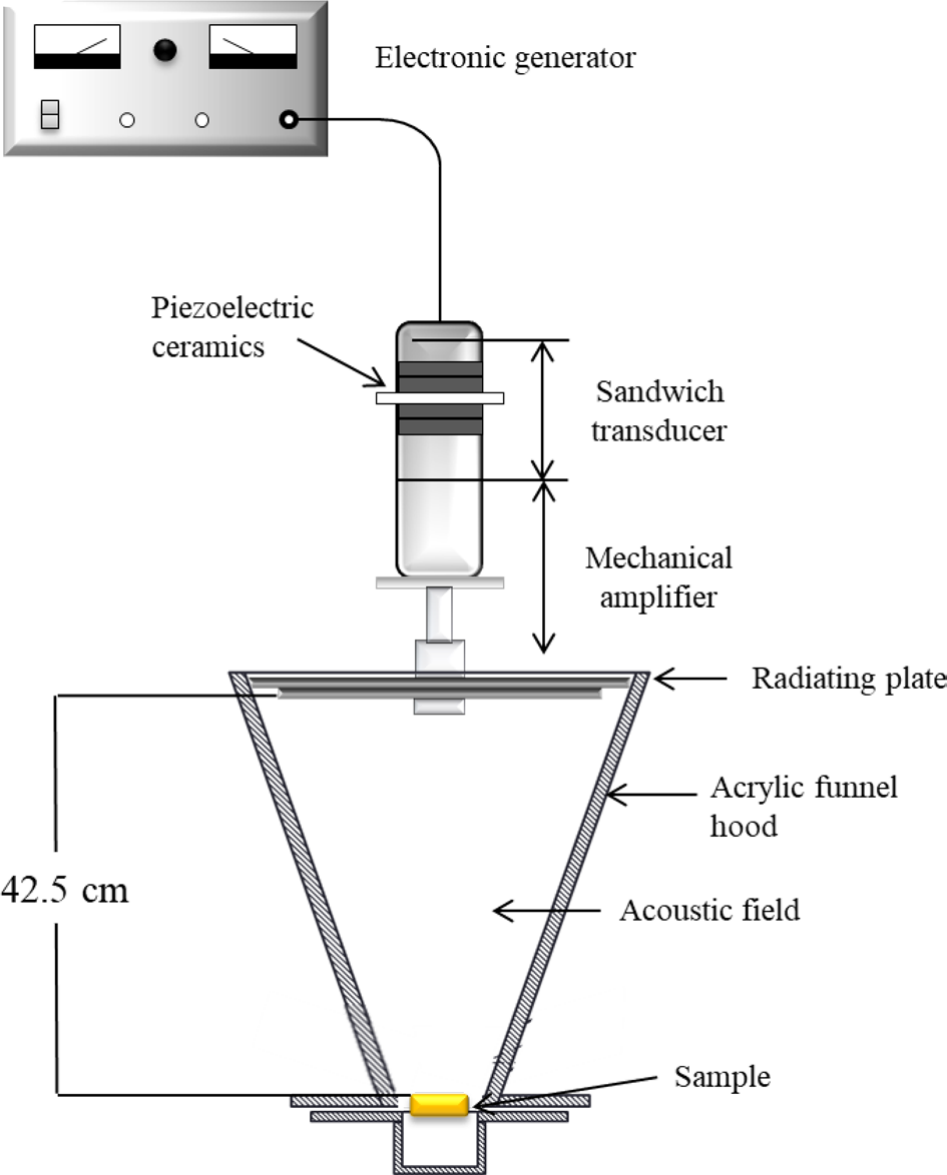
Schematic of the airborne acoustic ultrasound system.

All experiments were carried out using an airborne acoustic ultrasonic system (Pusonics S.L., Madrid, Spain) operating at a frequency of 26 kHz with a maximum power output of 170 W. The system consists of an electronic power generator with a dynamic resonance controller, a power amplifier, a high impedance matching box, and a circular stepped-plate transducer. The funnel-shaped acoustic airborne ultrasound construction provided highly focused acoustic field generated by the vibration of a plate transducer^44^. The biofilm coupons were placed on a stainless-steel plate positioned at a distance of 42.5 cm from the centre of the transducer at a maximum acoustic energy density of 10 W/cm^2^ (Fig. 4). The biofilm coupons were treated with airborne acoustic ultrasound for 15 min.

### Plasma activated water treatments

**Figure 5:**
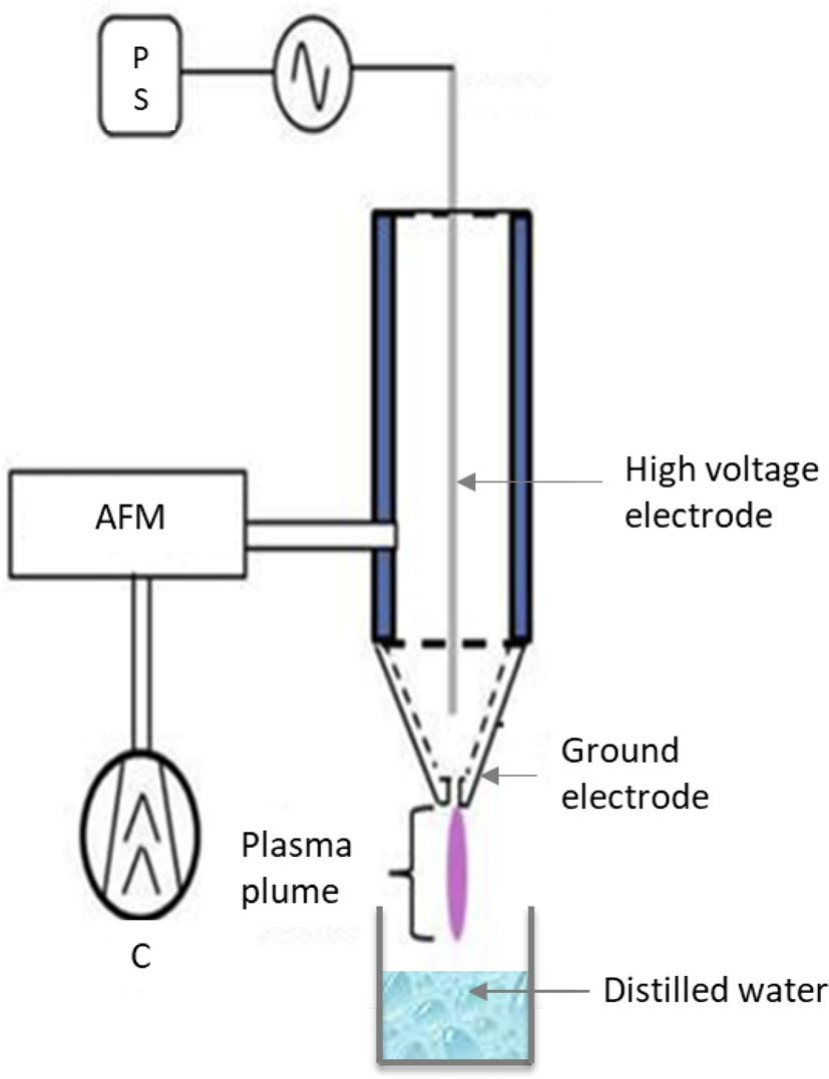
Schematic diagram of PAW generation by cold plasma jet system. (AFM) air flow monitor; (C) gas introduction; (PS) power supply.

Cold plasma treatments were carried out using a system sourced from the National Centre for Plasma Science and Technology at Dublin City University (Glasnevin, Dublin 9, Ireland).This system is elaborately described in previous publication^21^. Plasma generated within this region exited through a small gap in the ground electrode entering ambient conditions in the plume region where it came into contact with treatment samples (Fig. 5). Plasma was generated at 30 kV using ambient air as the working gas.

**Figure 6:**
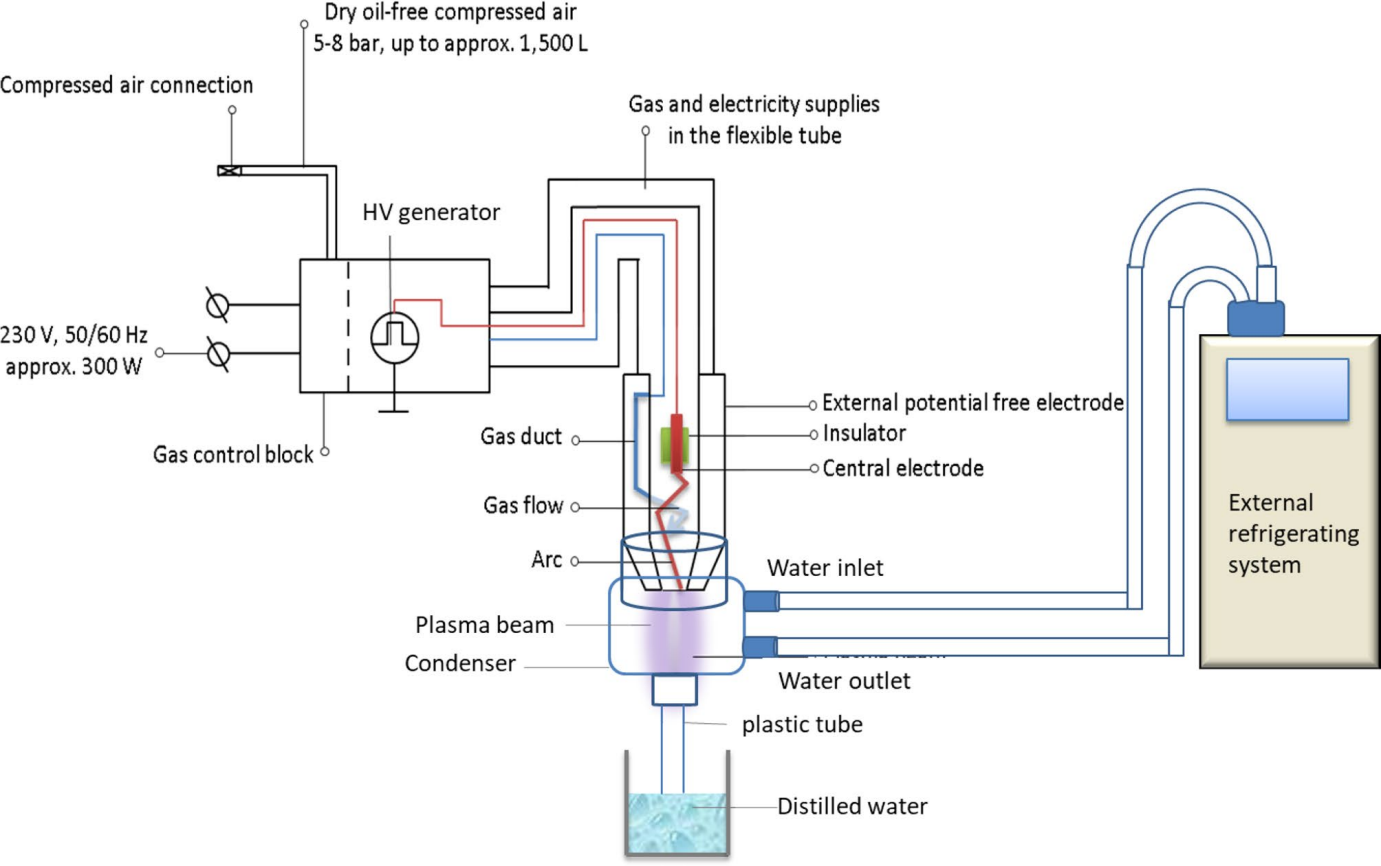
Functional principle of thermal atmospheric plasma system. HV: High voltage generator.

Thermal plasma treatments were carried out using a plasma beam system (Diener electronic GmbH & Co. KG, Ebhausen, Germany) operating at 20 kHz. The system was composed of three main units: a supply unit including a high voltage generator, plasma current and gas control devices; gas and power conductors in a flexible tube; and a plasma torch. The high voltage generator produced a voltage up to 10 kV, which was required to create the electric field to initiate and sustain the electrical plasma discharge. The electrical conductor for the supplied voltage and the process gas tubing were supplied to the plasma torch via a flexible tube, and the plasma particles (ions, electrons, excited atoms and molecules) were generated from the air supplied to the plasma torch. The temperature of the plasma gas would reach up to 200 °C; therefore, a condenser and cooling coil was used to maintain the temperature of the activated gas generated by the plasma at ambient temperature. Cold water (4 ± 0.5 °C) was circulated through the condenser using external refrigerating system (Lauda Ecoline, RE104). The functional principle of this system is shown in Fig. 6.

For PAW generation from thermal and cold plasma jet, the distance between the nozzle of the jet and water surface was around 7.2 cm. For generation of PAW, a Duran bottle containing 100 mL of sterile deionised water was treated with cold or thermal plasma for 15 min. Individual biofilm coupons were immersed in 10 mL of PAW for 30 min at 4 °C during treatments. Sterile distilled water was used instead of PAW for control treatments.

### Combined treatments

To determine the synergistic effects of combined treatment of airborne acoustic ultrasound and PAW, biofilm coupons were first exposed to airborne acoustic ultrasound as described in Sect. 2.3.1 followed by treatment with PAW as described in 2.3.2. Different treatment combinations were carried out as listed in Table 2.

**Table 2:** The pH, conductivity, ORP and RONS of PAW compared to untreated controls.

Parameters	Treatments		
	Control	Cold plasma	Thermal plasma
pH	6.71 ± 0.03	2.85 ± 0.18	2.53 ± 0.04
ORP (mV)	406.07 ± 12.7	565.40 ± 1.9	510 ± 0.6
Conductivity (µs/cm)	4.5 ± 0.5	333.00 ± 13.44	518.0 ± 22
Hydrogen peroxide (µM)	nd	14.7 ± 3.9	13.2 ± 5
Nitrate (µM)	nd	71.7 ± 5	13.09 ± 6
**Nitrite (µM)**	nd	33.5 ± 0.7	7 ± 0.2

Data are expressed as the mean value of three independent experiments, ‘nd’ indicates not detected.

### Microbiological analysis

The population of *E. coli* on control and treated coupons was enumerated using the plate count method. The biofilm cells were detached from coupons using glass beads as described by Vatanyoopaisarn^45^. The coupon was placed in sterile 10 mL of maximum recovery diluent (MRD, Oxoid Ireland c/o Fannin Healthcare, Ireland) with 10 sterile glass beads (18,406, Sigma-Aldrich, Ireland). After soaking for 10 min, the tube was subjected to vortex-mixing for 1 min at maximum speed, yielding a suspension of *E. coli* that adhered on the coupon surface. Decimal dilutions were then carried out and spread on nutrient agar plates. After 24 h incubation at 37 °C, the colony forming unit per millilitre (CFU/mL) was calculated.

### Confocal laser scanning microscopy

A live-dead BacLight bacterial viability and counting kit L34856 (Thermofisher Scientific, Ireland) was used. This included SYTO 9, a green fluorescent nuclear and chromosome counterstain, and propidium iodide (PI), a red-fluorescent stain which is not permeant to live cells, and thus was used to detect dead cells. The staining procedure was based on the instructions from the kit manufacturer with slight modifications. The working solution of fluorescent stains was prepared by adding 1.5 μL of SYTO 9 stain and 1.5 μL of propidium iodide stain to 1 mL of sterile 0.85% sodium chloride (NaCl). This working solution was prepared and used the same day. Each untreated and treated biofilm slide was covered with 200 µL of prepared staining solution and incubated at room temperature in dark for 15 min. The biofilm slides were carefully washed with 1 mL of NaCl solution twice to remove any dye residues. The biofilm samples were then placed on a 35 mm diameter glass-bottomed dish (ibidi GmbH, Martinsried, Germany) containing one drop of the mounting solution obtained from the live/dead BacLight bacterial viability kit L7007 (Thermofisher Scientific, Ireland). The slides were directly examined on a confocal laser scanning microscope (Olympus Fluoview FV1000) equipped with a 60x / 1.35 NA oil immersion objective. At least 3 randomly chosen microscopic fields were examined for each sample. A noise reduction filter was applied to the images in the Olympus Fluoview FV1000 software.

### Physicochemical properties of plasma activated water

The physicochemical properties (including pH, electrical conductivity, ORP, RONS) of PAW were measured immediately after plasma treatment. For these measurements, 30 mL of sterile distilled water was treated with either cold or thermal plasma for 5 min. The pH, electrical conductivity and ORP of each sample was measured using a pH meter (Hanna, pH 213, UK), an electric conductivity meter (Jenway, 4070, UK), and a redox sensitive electrode (Hanna, HI3230B, UK) respectively. Measurements of hydrogen peroxide (H_2_O_2_) generated in the PAW were determined by titanium oxysulfate calorimetric method; briefly 10 μL of TiOSO_4_ (Sigma-Aldrich, Ireland) was added to 100 μL of PAW and then incubated in the dark for 10 min. The titanium sulfonate reagent reacts with hydrogen peroxide present in PAW to produce coloured pertitanic acid, which was measured on spectrophotometric plate reader (BioTek Epoch 2C, Mason technology Ltd., Dublin, Ireland) at 390 nm. The levels of nitrite in PAW were measured using Griess reagent (N-(1-Naphthyl) ethylenediamine dihydrochloride) (Sigma-Aldrich, Ireland), where 50 µl of the reagent was added to 50 µL PAW and incubated in dark for 30 min. After incubation, absorbance of samples was measured at 548 nm. When detecting nitrate concentration, PAW was pre-treated with sulfamic acid to eliminate nitrite interference. Nitrate concentration was then assessed by 2,6-dimethyl phenol (DMP) using the Spectroquant nitrate assay kit (Merck Chemicals, Darmstadt, Germany) with manufactures instructions. During each reactive species assessment, a known standard was included in each plate to convert absorbance values into known concentrations.

### Statistical analysis

All experiments were performed in three independent experiments with three technical replicates each. Analysis of variance (ANOVA) and Tukey’s test was carried out using SAS (Version 8.0) and considered statistically significant at *p* < 0.05. The parametric assumptions were verified before performing ANOVA: normal distribution (Shapiro–Wilk test), homogeneity of variances (Levene test) and data independence (by experimental design).
